# Multidimensional Model of Environmental Attitudes: Evidence Supporting an Abbreviated Measure in Spanish

**DOI:** 10.3390/ijerph18094438

**Published:** 2021-04-22

**Authors:** Elena Andrade, Gloria Seoane, Luis Velay, Jose-Manuel Sabucedo

**Affiliations:** 1 CRETUS Center, Department of Social Psychology, Basic Psychology and Methodology, Faculty of Psychology, Universidade de Santiago de Compostela, 15782 A Coruña, Spain; mgloria.seoane@usc.es (G.S.); luismiguel.velay@rai.usc.es (L.V.); josemanuel.sabucedo@usc.es (J.-M.S.); 2 CFR Pontevedra, Consellería de Educación, Universidade e Formación Profesional, 36003 Pontevedra, Spain

**Keywords:** environmental attitude, measurement, abbreviated model, validity, sustainability, culture

## Abstract

We conducted three independent studies to support the Spanish version of the Environmental Attitudes Inventory (EAI). The first study consisted of translating and pre-testing on a sample of 125 college students. The second consisted of testing the EAI on a sample of 225 university students in several undergraduate courses. Student data were collected using two different methods, through an online teaching platform and in the classroom. The findings were symmetrical in terms of precision and dimensionality. The third study completed the aforementioned ones testing the items on a representative sample from the general population in Spain. The participants were 630 citizens from 17 regions and responded to the EAI using an online platform. The results of the factor analysis led us to propose a measurement model, with 18 items and six first-order factors: environmental movement activism, conservation motivated by anthropocentric concern, confidence in science and technology, personal conservation behaviour, human dominance over nature, and support for population growth policies. External validity evidence was assessed by the correlation with the following variables: neuroticism, ecological behaviour, limits to economic growth, economic liberalism, sustainability, altruism, and social desirability. These estimations stayed away from demographic and personal aspects such as age, sex, political ideology, and region.

## 1. Introduction

Understanding people’s behaviour in their environment, whether natural or constructed, involves studying how individuals assess such an environment [1], i.e., it implies an effective evaluation of psychological variables, including attitudes. It seems accepted that attitudinal change affects behaviour [2]. However, knowledge of attitudes will depend on the quality of the instruments used for measurement [3].

### 1.1. Tradition in Environmental Attitude Assessment

Attitudes towards the environment are often revealed through explicit methods such as self-reports [4]. To advance research in this field, the authors preferred to create new instruments rather than progress from theoretical development and the properties of existing ones [5]. This tendency entails difficulties, such as the lack of integration of results, in the sense of being able to establish the relative importance of attitudes on behaviour [6].

At least 700 measures of attitudes towards the environment have been documented [7]. Most of them have a multidimensional character, and they use a response format of between four and seven points. They have been tested in Anglo-Americans, with very few exceptions, such as the Environmental 2-MEV Scale [8], which was initially designed for European children and adolescents. 

The instrument that may appear most often in the literature on environmental issues has been the New Environmental Paradigm (NEP) or its revised version, The New Ecological Paradigm [9]. Nevertheless, it is a scale that caters only to general aspects of the relationship between the individual and environment. It reflects the extent to which a person believes that humans are part of nature rather than being independent or superior to it (ecocentrism vs. anthropocentrism). In addition, this instrument has been applied with a variable number of items, of differing content and several answer formats [6].

### 1.2. Recent Approaches and the Theory of Ecological Attitude

A recent, more comprehensive measure is the Environmental Attitudes Inventory [10]. To build it, the authors based it on several previous relevant models, among them, the NEP. As opposed to the classic idea of attitude, with three components, evaluative, affective, and behavioural, Milfont and Duckitt defend a contemporary conception of attitudes towards the environment. They define them as psychological tendencies to evaluate the environment with a certain level of liking or displeasure. Beliefs, affection, and behavioural intentions are not components, but bases that are influenced upon by attitudes and from which attitudes can be inferred [4].

As for dimensionality, Milfont and Duckitt [10] adhere to the prevailing idea that the construct of attitudes towards the environment is multidimensional. From a theoretical point of view, it has also been suggested that the first-order dimensions can be organized in a hierarchy with two dimensions of a higher order, called preservation and utilisation. In this way, they adopt the theory of ecological attitude, formalised by Wiseman and Bogner [11]. 

According to the theory of ecological attitude, nature preservation would be a biocentric dimension, which reflects the conservation and protection of the environment. Meanwhile, utilisation would be an anthropocentric dimension, which contemplates the exploitation of natural resources [11]. As opposed to the NEP model, both dimensions on environmental perception are different in this case and not poles of the same continuum [3].

The Environmental Attitudes Inventory (EAI) is a model made up of 12 scales, which highlight the following aspects: (1) enjoyment of nature, (2) support for interventionist conservation policies, (3) environmental movement activism, (4) conservation motivated by anthropocentric concern, (5) confidence in science and technology, (6) environmental fragility—threat, (7) altering nature, (8) personal conservation behaviour, (9) human dominance over nature, (10) human utilisation of nature, (11) ecocentric concern, and (12) support for population growth policies.

Each scale includes 10 statements. Seven of the proposed scales (1, 2, 3, 6, 8, 11, and 12) represent the second-order factor preservation. Five other scales (4, 5, 7, 9, and 10) represent the second-order factor utilisation. An example of an item relative to preservation would be, according to the theory of ecological attitude, “Being out in nature is a great stress reducer for me”. An example of an item relative to utilisation would be “Humans were meant to rule over the rest of nature”.

As a target, the EAI aims, therefore, to capture the multidimensionality and the hierarchical structure of attitudes towards the environment. It has been applied in different cultural contexts, such as in the population of New Zealand, the United States, Brazil, and South Africa [10] and, more recently, in France [12] and Portugal [13]. It is also being prepared for use in Malaysia [14].

### 1.3. The Nomological Network

As for the nomological network of relations, a different profile is foreseen for the components of preservation and utilisation. Wiseman and Bogner [11] reported a disparate association with personality traits. Preservation would be associated in a positive way with neuroticism, and utilisation with psychoticism.

Previous empirical evidence has shown significant correlations of preservation with ecological behaviour. Equally, it has shown higher correlations with variables such as limits to economic growth, sustainability, and social desirability. Utilisation seems to better predict economic liberalism [10,15].

A positive relationship is also expected from the biocentric dimensions of attitude (i.e., factors of preservation) with “self-transcendence’s nonenvironmental values” such as altruism [16,17,18].

With regard to the link between attitudes towards the environment and socio-demographic variables, findings are diverse. Although belonging to the younger population, being a woman, having a high level of education, having less conservative ideology, and living in urban areas are characteristics that have been linked to a better attitude towards the environment [5,19,20,21].

The results are promising, even though Milfont and Duckitt [10] did not study the relationship between first-order dimensions of attitudes and external variables in detail. Nonetheless, the authors of the EAI admit that the vertical structure, with preservation and utilisation, has not yet been proven. Preservation and utilisation overlapped to a great degree and did not predict ecological behaviour individually to a greater extent than the joint attitude score. In this fashion, both the hierarchical structure and the meaning through external variables of the dimensions posed require further investigation.

### 1.4. Economy in Applied Contexts and Cultural Issues

The EAI, on the other hand, is originally an excessively long questionnaire. Its complete form contains 120 items and can be applied with 72 items, if only six are administered for each of the 12 scales. As a consequence of such a broad number of items, response rates and completion may be affected, especially when participants feel they have to respond to multiple expressions of the same belief or behaviour [22].

Several shorter forms of the EAI have been proposed. The 24-item version by Milfont and Duckitt [10], although interesting, contains only two items per factor, and an item is the reverse statement of the other. The reduced version by Sutton and Gyuris [22] contains 37 items compared to the original 72, which were selected based on criteria of internal consistency, item-total correlations, normality, balance between items formulated directly and inversely, and sensitivity in change detection as a result of the implementation of environmental education programs.

Sutton and Gyuris [22] compared 37 items with the original 72 from the EAI in a population of university students in their first and last years. They concluded that the shorter form was a reliable tool with certain validity to investigate attitudes towards the environment in university students. At the same time, it reduced fatigue and increased response rates compared to longer versions of the EAI. 

The investigation of Sutton and Gyuris [22] contributed to establishing the EAI as a “gold standard” to monitor students’ environmental attitudes. However, the analysis of this model’s psychometric properties measured in terms of dimensionality and external tests of validity also need expanding.

Moreover, it is difficult to assume that the interpretation of scores can be generalised from one culture to another. Hence the reason for this work, focussed on testing how the items work in our context, and to provide an adapted Spanish version of the EAI. 

## 2. Method

Taking the proposal by Sutton and Gyuris [22], we perform a translation and three independent empirical tests of the translated instrument on the Spanish population. With these sequential tests, we fundamentally sought to contribute proof of reliability and internal and external proof of validity.

The first and second studies tested the instrument on two independent college student samples. The sampling method was non-probabilistic. Besides, student data were collected using two different methods, through an online teaching platform and in the classroom. The third study advanced the aforementioned ones testing the items on a representative sample from the general population in Spain. The type of sampling was probabilistic, and the participants responded to the EAI using an online platform. In all studies the statistical methods involved internal consistency coefficients, exploratory and confirmatory factor analyses, and application of the general linear model to assess the effect of the external variables. Besides, in the third study we used a multilevel model to estimate the variability in environmental attitude ascribable to the region of residence.

This project received the approval of the Bioethics Committee of the University.

**Study 1. Translation and pre-testing.**

## 3. Materials and Methods

### 3.1. Participants

A sample of 125 undergraduate students in psychology took part in the conventional pre-test, all of them from the same public university in northern Spain. Women made up 84.8%, and 15.2% were men. Ages were between 18 and 34 years old (*M* = 19.06, *SD* = 2.37). On a scale of 0 to 10, their perceived level of physical health was 7.17 (*SD* = 1.32). A mean of 4.02 h per week were dedicated to sports (*SD* = 5.95; *range* = 45–0), and 80% of the sample declared themselves as non-smokers (tobacco consumption *range* = 12–0). 

With regard to their political preferences, 79.2 identified themselves with the left wing or moderately left wing, 9.6% with the centre, and 11.2% with moderate right wing or right wing.

### 3.2. Instruments

#### 3.2.1. Environmental Attitudes Inventory

A direct and inverse translation was made of the Sutton and Gyuris [22] EAI version. This form of the test had 37 items, relating to the 12 dimensions or original factors. The translation task was undertaken by a small team made up of the authors of this article, with knowledge in measuring attitudes as well as language skills in Spanish and English, and by a native English speaker, with training in English philology and a good understanding of both the Spanish and British cultures.

A set of nine statements was tested in a personal interview, within the framework of a broader study of the research group. The items represented factors labelled as environmental movement activism, conservation motivated by anthropocentric concern, and confidence in science and technology. They were selected by their content and face validity as conceptually relevant for the purpose of study. A minimum of 57 people responded voluntarily to these items. As regards its qualitative contributions, it should be noted that statements formulated inversely were more difficult to respond to. Doubts were also raised about the meaning of long and complex items (e.g., “The belief that advances in science and technology can solve our environmental problems is completely wrong and misguided”, representing dimension number 5).

During adaptation we attempted to be as clear as possible and avoid negations. Items were responded to on a scale of 1 to 7. All the responses were labelled accordingly, with the following equivalences: 1 = strongly disagree, 2 = disagree, 3 = somewhat disagree, 4 = neither agree nor disagree, 5 = somewhat agree, 6 = agree, 7 = strongly agree.

#### 3.2.2. Neuroticism

The study also selected six items from the scale of neuroticism–emotional stability of the Revised NEO Personality Inventory (NEO PI-R), revised for Spanish by Cordero, Sanjeev, and Seisdedos [23]. Examples of the items used were: “I’m quite stable emotionally”, “Sometimes I do things impulsively and later I regret it”. The coefficient of reliability in this sample was 0.73.

#### 3.2.3. Demographic and Personal Variables

Together with items in the EAI and neuroticism, participants answered demographic questions, such as sex and age, and questions about their perceived level of physical health (on a scale from 1, very low, to 10, excellent), weekly time dedicated to sport, and daily cigarette consumption. They were also asked to identify what political option (left, moderate left, centre, moderate right, or right) better represented their ideas.

### 3.3. Procedure

Participants responded to the questionnaire as part of their daily activity on the university’s online campus. The online environment was accessed with their own usernames and passwords. Participants were guaranteed anonymity in their answers and were told they would be used for research purposes only. The data were recorded over one week, in the first month of the academic year.

## 4. Results

Fourteen items of attitudes and three of neuroticism were conveniently recoded, since they had an inverse sense. Data were analysed with the IBM SPSS Statistics Package 24 and with the LISREL 9.30 program.

### 4.1. Internal Consistency and Dimensionality of Attitudes

Calculated from the original composition, the alpha coefficient of internal consistency was positioned between 0.29 and 0.84. The lowest values corresponded to the factors abbreviated as Factor 2 (0.29), Factor 6 (0.37), Factor 11 (0.42), Factor 7 (0.54), Factor 10 (0.54), and Factor 4 (0.63).

As it was the first empirical test to be translated in this way, we performed an exploratory factor analysis (EFA) to study the dimensionality underlying correlations among the 37 items. The statistician values of Kaiser, Meyer, and Olkin (KMO = 0.711) and of Bartlett’s test for sphericity (*X*^2^(666) = 1836.923, *p* < 0.001) were acceptable. Although several forms of extraction and rotation were run, we preferred to use the principal axis method of factor extraction (recognising, thus, the unique component of variance of each item) and by the direct oblimin rotation (since it was anticipated that the factors would be to some extent correlated). 

A structure composed of 12 factors managed to explain 54.95% of the variance. However, only six of the original factors emerged clearly and defined by their three items. These factors were previously numbered 1, 3, 5, 8, 9, and 12. The solution choosing only the items relative to these factors explained 59.35% of the variance, the first six had an initial eigenvalue greater than 1. The factorial coefficients were all above 0.40, and three items (in Factors 3 and 5) showed a correlation close to 0.27 with a second factor.

The result with six factors accurately replicated the first-order structure expected for the 18 items deemed most stable. However, second-order factorisation did not provide support for superior preservation and utilisation factors. Correlations between factors were relatively low and reached 0.30 in only two cases. 

Complementarily, and in order to provide a first assessment of fit between this model and the data, a confirmatory factor analysis (CFA) was performed. We used the robust maximum likelihood method [24], since the assumption of multivariate normality was not complied with. The results for the general descriptive indices were as follows: SBX^2^(120, *N* = 125) = 168.84, *p* < 0.0022, CFI = 0.95, NNFI = 0.93, RMSEA = 0.057 (90% CI [0.035, 0.077]), and SRMR = 0.083. 

With such a small number of responses, estimates of the parameters were not very precise, with high typical errors. However, they are shown in Table 1, along with descriptive statistics and internal consistency coefficients for the six factors that make up this model extracted from the pre-test. The global value of alpha was 0.68.

### 4.2. Relationship with Personal and Demographic Variables

The global mean score in the EAI was calculated, and the partial averages were obtained for the six factors of the proposed model. It should be noted that no significant relationship was found between the scores in attitude towards the environment and the variables age, perceived level of physical health, weekly sports practice time and tobacco use. 

Nor did we find any differences in the global value of attitude on the basis of sex. Although the group made up of women significantly surpassed the group of men in Factor 3, activism (*F*(1, 123) = 6.60, *p* = 0.01; *Mwomen* = 5.25, *SD* = 1.10; *Mmen* = 4.54, *SD* = 1.13). The differences in Factors 9 and 12 were relatively high, without reaching statistical significance.

As for political ideology, it was not associated to age or sex of participants. Nor was it associated to the general attitude towards the environment. We did find, instead, an effect of the political option in relation to Factor 9, human dominance over nature (*F*(3, 121) = 4.73, *p* = 0.004). Because the size of the groups based on this variable was unbalanced, in the analysis the responses of the participants were divided into just left, moderate left, centre, and right (which grouped moderate right and right). The *a posteriori* contrasts of Bonferroni revealed that the difference was significant between left (*n* = 58) and right (*n* = 14): *M*_1_ – *M*_4_ = 1.10, *p* = 0.004, and also between moderate left (*n* = 41) and right (*n* = 14): *M*_2_ – *M*_4_ = 0.99, *p* = 0.017. Here, the left and moderate left groups expressed a more positive attitude towards the environment.

### 4.3. Relationship with Neuroticism

Finally, we found a significant relationship between global attitude and external measure of neuroticism: *F*(2, 122) = 9.53, *p* < 0.001; *R*^2^ *adjusted* = 0.12. Adjusting for the sex variable, we obtained partial correlations for both this global score in attitude as well as for two factors (*F*(2, 121) = 8.96, *p* < 0.001; *R*^2^ *adjusted* = 0.16): Factor 12, support for population growth policies (*r partial* = 0.27, *p* = 0.003) and, to a lesser extent, for Factor 5, confidence in science and technology (*r partial* = 0.19, *p* = 0.034). The tolerance values for these analyses were placed between 0.960 and 0.985.

**Study 2. Testing in university students.**

## 5. Materials and Methods

### 5.1. Participants

The sample was also “convenient” and made up of 208 psychology students from the same university, located in the north of Spain. Second year students made up 33.7% and 38.9% were in the third and 27.4% in the fourth year. 

Women participants made up 80.3% and 19.7% men. Mean age was 21.11 years (*SD* = 2.21; *range* = 36–19). On a scale of 0 (very low) to 10 (excellent), perceived level of physical health was 7.04 (*SD* = 1.25). They devoted an average of 3.28 h per week to sport (*SD* = 2.78; *range* = 14–0). Of the sample, 84.6% declared themselves non-smokers (*tobacco consumption range* = 20–0).

### 5.2. Instruments

#### 5.2.1. Environmental Attitudes Inventory

The EAI was applied in full, with the 37 items of the first empirical study, to test if results were replicated in a wider sample.

#### 5.2.2. External Variables

As part of the same document, and in order to obtain external evidence of validity, participants responded to the following measures of external variables. The choice of these variables is preceded by its demonstrated convergence or discrimination with respect to the EAI in other cultural contexts [10,12,15,22].

The measure of ecological behaviour was small test of eight items, used by Schultz, Zelezny, and Dalrymple [25] with students of Social and Behavioural Sciences in Spanish speaking countries. Participants noted the frequency with which they had carried out in the last year eight actions of environmental care (e.g., recycling of cans or bottles, purchase of products in reusable or recyclable containers). To do this, a scale with five categories of response was used: 1 = never, 2 = rarely, 3 = sometimes, 4 = often, 5 = very often. 

Measures of economic liberalism, limits to economic growth, and sustainability were integrated with the items of the EAI, in the same seven-point response format.

The scale of economic liberalism consisted of three items initially proposed to evaluate the economic dimension of the dominant social paradigm [26]: “Individual behaviour should be determined by economic self-interest, not politics”, “If the economy continues to grow, everyone benefits”, and “The best measure of progress is economic”.

As an indicator of limits to economic growth, an item derived from the NEP was incorporated: “There are limits to economic growth beyond which our industrialized society cannot expand”.

Finally, we also included a statement along the lines of the classic definition of sustainability [27], often referred to in the literature: “In the development of our society we must strive to meet the needs of the present without compromising the ability of future generations to meet their own needs”.

#### 5.2.3. Abbreviated Scale of Social Desirability

It consisted of 10 balanced items belonging to the dimension of impression management from the Balanced Inventory of Desirable Responding (BIDR). The Spanish versions were published by Moral de la Rubia, Garcia-Cadena, and Antona-Casas [28] and by Mikulic, Crespi, and Caballero [29]. Two examples of items would be: “I always obey laws, even if I’m unlikely to get caught”, “I have never dropped litter on the street”. The response format consisted of seven sorted categories, with values 1, 4, and 7 labelled: not true, somewhat true, and very true, respectively.

#### 5.2.4. Demographic and Personal Variables

Each participant’s year and the usual variables of sex and age were recorded. Additionally, for this sample they were requested to report on their perceived level of physical health (on a scale of 1, very low, to 10, excellent), weekly time dedicated to sport, and daily cigarette consumption. Finally, they indicated which political option best represented their ideas. In this case, the item was expressed as is commonplace in studies by the Spanish Centre for Sociological Research [30], with a graduated response between 1 (left) and 10 (right).

### 5.3. Procedure

Students completed the document relating to the EAI and other variables at the beginning of a daily class session. The application was carried out with the permission of the professor in charge of the class, and all the participants signed an informed consent for anonymous use of their answers for research purposes.

## 6. Results

The data were analysed using the statistical package IBM SPSS Statistics 24 and the LISREL 9.30 program. The analyses were carried out with the complete responses of 206 participants. From the original structure, with 12 dimensions, we calculated the Cronbach alpha coefficient, which showed values between 0.44 and 0.87. The following theoretical factors presented unsatisfactory coefficients: Factor 2 (0.44), Factor 4 (0.48), Factor 6 (0.53), Factor 7 (0.52), Factor 10 (0.8), and Factor 11 (0.55).

We conducted EFA and CFA with criteria similar to those used in Study 1. EFA solutions led us to decide to maintain the structure with the same six first-order factors. Nor in this case do we find favourable evidence for higher order postulated factors. According to the principal axis method of factor extraction and with oblique rotation, the solution corresponded with that obtained in the first sample and explained 56.69% of the variance. 

The model was submitted to CFA. On the right-hand side of Table 1 you can see the results for this second study in terms of general descriptive statistics, CFA model estimates, and reliability. The alpha value for the whole of the 18 items was 0.75.

For the CFA, the robust maximum likelihood method was used once again. The global adjustment values were as follows: SBX^2^(120, *N* = 206) = 147.73, *p* < 0.044, CFI = 0.98, NNFI = 0.98, RMSEA = 0.034 (90% CI [0.0062, 0.050]), and SRMR = 0.054. All the factor–item relationship estimates and error variances were significant. Standardised residuals showed values between −2.89 and 2.71.

We obtained significant correlations between Factors 1 (enjoyment of nature), 3 (environmental movement activism), 8 (personal conservation behaviour), and 9 (human dominance over nature). The largest overlap is found between Factors 3 and 8 (φ = 0.71). As for the other coefficients, they were higher for the relationship of Factor 1 with Factor 8 (φ = 0.47), with Factor 3 (φ = 0.46) and with Factor 9 (φ = 0.34). The correlations of Factor 12 (support for population growth policies) with others were not significant. Factor 5 (confidence in science and technology) was only related to Factor 9 (φ = 0.17). 

### 6.1. Relationship with Demographic and Personal Variables

Once more we calculated the global and partial averages in attitude towards the environment. The correlation between participants’ attitude and age was not significant, nor with their perceived level of physical health, time dedicated to sport, nor with tobacco consumption. 

We did not find a statistical effect of sex on the global average in attitude, but did find a significant effect on Factor 5 (*F*(1, 53.731) = 6.77, *p* = 0.012), Factor 12 (*F*(1, 206) = 9.141, *p* = 0.003), and Factor 9 (*F*(1, 206) = 4.002, *p* = 0.047). The group made up of women showed a higher score in Factors 5 (*Mwomen* = 4.20, *SD* = 1.18; *Mmen* = 3.58, *SD* = 1.44) and 9 (*Mwomen* = 6.11, *SD* = 0.90; *Mmen* = 5.79, *SD* = 1.02); and lower in Factor 12 (*Mwomen* = 2.61, *SD* = 1.19; *Mmen* = 3.26, *SD* = 1.36).

In this sample, political ideology was again independent of sex and age and only showed a marginal correlation with attitude towards the environment (*F*(1, 202) = 3.59, *p* = 0.059; *R*^2^ *adjusted* = 0.01). Describing in detail, Factor 9 proved to be the best predictor of the value of the political scale (*F*(1, 202) = 9.504, *p* = 0.002; *R*^2^ *adjusted* = 0.04; β = −0.21; *r partial* after controlling for sex = −0.21). Participants located on the far right of the scale tend to have a less positive attitude towards nature. 

There were no differences in attitude towards the environment scores in relation to the year to which the students belonged.

### 6.2. Relationship with External Variables

We performed separate linear regression analyses of external variables (see Table 2) on the overall score and also on partial scores of attitude towards the environment. For each case, we incorporated the possible effect of socio-demographic variables to the model.

Ecological behaviour. Overall attitude and age were significantly associated with Ecological behaviour ((*F*(2, 205) = 26.32, *p* < 0.001; *R*^2^ *adjusted* = 0.20; *r partial* for attitude = 0.40). Factor 8 (β = 0.50, *p* < 0.001; *r partial* = 0.51) and Factor 3 (β = 0.22, *p* < 0.001; *r partial* = 0.25), followed by Factor 5 (β = −0.12, *p* = 0.02; *r partial* after adjusting of age and sex = −0.17) were the best predictors of this variable (*F*(4, 201) = 42.77, *p* < 0.001; *R*^2^ *adjusted* = 0.45).

Economic liberalism. Attitude was associated in a significant and negative way with economic liberalism, whereas political tendency showed a significant and positive relation (*F*(2, 201) = 20.62, *p* < 0.001; *R*^2^ *adjusted* = 0.16; *r partial* for attitude = −0.35). Factor 9 (β = −0.31, p < 0.001; *r partial* after controlling for political ideology and sex = −0.33) and Factor 3 (β = −0.19, *p* = 0.003; *r partial* = −0.21) predicted this variable of economic liberalism significantly (*F*(3, 200) = 16.37, *p* < 0.001; *R*^2^ *adjusted* = 0.19).

Limits to economic growth. Global attitude and political tendency predicted scoring in limits to economic growth (*F*(2, 201) = 15.11, *p* < 0.001; *R*^2^ *adjusted* = 0.12; *r partial* for attitude = 0.32). Factor 12 was the attitude component that predicted participants’ opinions about setting limits on economic growth (*F*(2, 201) = 9.68, *p* = 0.001; *R*^2^ *adjusted* = 0.08; β = 0.24, *p* < 0.001; *r partial* after controlling for political ideology and sex = 0.26).

Sustainability. Neither the overall score nor the partial measures of attitude significantly predicted opinion on sustainability.

Social desirability. Global attitude did not show a significant linear association with the scale of social desirability. Factor 8 was the only measure of attitude with some kind of effect on this external variable (*F*(1, 205) = 6.79, *p* = 0.01; *R*^2^ *adjusted* = 0.03; β = 0.18).

**Study 3. Validity evidence in general population.**

## 7. Materials and Methods

### 7.1. Participants

The sample was made up of 630 Spanish citizens. They were chosen by proportional random sampling of 17 autonomous communities and respecting the balance in the representation of the variables sex and age. Fifty percent of the participants were men and 50% women. Mean age was 44.16 years (*SD* = 14.46; *range* = 80–18).

### 7.2. Instruments

#### 7.2.1. Environmental Attitudes Inventory

The EAI was used again, with 37 items, relative to 12 conceptual dimensions, with a minimum of three items per dimension. Several items were reformulated, because the expected results for several inventory factors had not been obtained in the previous tests.

#### 7.2.2. External Variables

In addition to the EAI, participants completed the ecological behaviour test, whose score could range from 1 to 5, as well as the previously mentioned measures of economic liberalism, limits to economic growth, and sustainability. The latter had values between 1 and 7. They were also given the abbreviated scale of social desirability, with scores between 1 and 7. 

Finally, they completed an altruism test [16]. In it they had to judge the importance of a small list of values “as a guide for their own life”: Equality, a world in peace, social justice, and helpful (working for the welfare of others). Therefore, there were four items, which quantified the answers: opposed to my values as zero, not important as 1, and extremely important as 9.

#### 7.2.3. Demographic and Personal Variables

In the same way, each participant indicated their sex, age, autonomous community, and even the classification of the type of area based on the criteria rural versus urban and inland versus coast.

Finally, they indicated which political option best represented their ideas, according to the scale of the Spanish Centre of Sociological Research [30], scaled between 1 (left) and 10 (right).

### 7.3. Procedure

The data were collected through the online platform *Qualtrics*. Participants were informed that in answering and submitting the questionnaire, they gave their consent to being included in the study. Anonymity and confidentiality were fully guaranteed, so that individual responses would not be known or used.

## 8. Results

The data were analysed using the statistical package IBM SPSS Statistics 24, the LISREL 9.30 program, and the lm4 program in R.

We obtained Cronbach’s alpha values for the 12 proposed dimensions of attitude. The so-called Factor 2 (0.45), Factor 6 (0.33), and Factor 7 (0.29) showed very low internal consistency. For the rest of the dimensions evaluated, we recorded a minimum value of 0.64, shared by Factors 1, 4, and 5, and the maximum value of 0.85, corresponding to Factor 12.

We tested the stability of the factorial structure through several exploratory as well as confirmatory methods. Although we intended to retain as many dimensions as possible, no solution with more than six factors became statistically satisfactory. The best factorial composition consisted of the six first-order factors (five of them common to studies 1 and 2) described as 3 (environmental movement activism), 4 (conservation motivated by anthropocentric concern), 5 (confidence in science and technology), 8 (personal conservation behaviour), 9 (human dominance over nature), and 12 (support for population growth policies).

Conceptually, Factors 3, 8, and 12 would be closer to the second-order dimension of preservation. On the other hand, Factors 4, 5, and 9 better represent the theoretical dimension of utilisation. However, the adjustment of the aforementioned second-order structure was inadequate. In fact, it was not possible to identify a second-order structure in accordance with theoretical predictions.

Table 3 lists the statistics corresponding to the solution chosen after analysing the data from this sample of the general population. The table also contains the internal consistency values. Worthy of note is the change of Factor 4 for Factor 1 in this proposal. Although the internal consistency of both factors was similar, the homogeneity of the items, the global adjustment of the model, and the study of the residuals favoured the decision to keep Factor 4. As regards Factor 5, its internal consistency coefficient is lower than in Studies 1 and 2. Nevertheless, this factor emerged in all three studies as a statistically and conceptually different dimension. The value of alpha considering the whole of the six factors, with 18 items, was 0.73.

The CFA results for this model were as follows: SBX^2^(120, *N* = 630) = 452.92, *p* < 0.001, CFI = 0.95, NNFI = 0.94, RMSEA = 0.066 (90% CI [0.060, 0.073]), and SRMR = 0.079. All estimates of the factor–item relationship were significant, with typical errors between 0.05 and 0.08. 

The pattern of correlations between factors was similar to that of student samples, though, as expected, of greater intensity. We obtained the highest correlations between Factor 4 (conservation motivated by anthropocentric concern) and Factor 3 (environmental movement activism), with φ = 0.74; between Factor 4 and Factor 8 (personal conservation behaviour), with φ = 0.78. Factors 3 and 8 also correlated significantly: φ = 0.58. The following, in order of magnitude, were the correlations of Factor 9 (human dominance over nature) with Factors 5 (confidence in science and technology) and 12 (support for population growth policies), with values of φ = 0.47 and φ = 0.35, respectively. Factor 12 correlated with Factor 5 (0.25), as well as with Factor 3 (0.24) and with Factor 4 (0.17). Factor 9 correlated with Factor 8 (0.22) and with Factor 4 (0.15). Factor 5 showed a marginally significant correlation with Factor 3 (0.10).

### 8.1. Relationship with Demographic and Personal Variables

We obtained the global and partial averages in attitude towards the environment. Through a multilevel analysis (using the *lmer* function of the *lm4* program in R), we found that the variation in attitude towards the environment attributable to the autonomous community of residence was negligible; under 1% in the overall score and with a maximum of 0.01% when considering the different factors. Consequently, the following analyses were carried out using conventional regression and analysis of variance options.

Neither the overall score in attitude nor the partial measures were significantly associated with the type of residence area.

The mean score in attitude did not correlate with the age of participants. According to partial measures of attitude, we obtained a positive correlation of age with the score in Factor 5 (*r* = 0.183, *p* < 0.001) and in Factor 8 (*r* = 0.171, *p* < 0.001), and a negative correlation with Factor 12 (*r* = −0.178, *p* < 0.001).

In this sample we did not find a significant effect of sex on the global average in attitude, either. After statistically controlling age, we found significant differences between women and men in Factor 9 (*F*(1, 627) = 8.288, *p* = 0.004) and in Factor 12 (*F*(1, 627) = 5.882, *p* = 0.016). The mean in Factor 9 was higher for women (*Mwomen* = 4.97, *SD* = 1.56; *Mmen* = 4.60, *SD* = 1.56). The mean in Factor 12 was higher in the men’s group (*Mwomen* = 3.33, *SD* = 1.59; *Mmen* = 3.77, *SD* = 1.63).

Political ideology was independent of sex and age of participants. It correlated significantly with the global attitude towards the environment score, although the effect size was low: *F*(1, 628) = 28.73, *p* = 0.001; R^2^ *adjusted* = 0.042; β = −0.21. Again, Factor 9 proved to be the best predictor of the value of the political scale (β = −0.27, *p* < 0.001; *r partial* after controlling for sex = −0.26), accompanied by Factor 12 (β = −0.12, *p* = 0.004; *r partial* after controlling for sex and age = −0.11): *F*(2, 627) = 22.84, *p* = 0.001; R^2^ *adjusted* = 0.07).

### 8.2. Relationship with External Variables

As in Study 2, we performed a linear regression analysis of external variables (see Table 4) on the global and partial scores of attitude towards the environment. The possible effect of socio-demographic variables, which in all cases was weak, was also taken into account.

Ecological behaviour. On a global level, the average of attitude and age were significantly associated with the behavioural self-report: *F*(2, 627) = 65.53, *p* < 0.001; *R*^2^ *adjusted* = 0.17; *r partial* for attitude = 0.41. On a specific level, the effect of age ceased to be significant. As in Study 2, Factor 8 (β = 0.33, *p* < 0.001; *r partial* after controlling for age = 0.30), Factor 3 (β = 0.25, *p* < 0.001), and to a lesser extent, Factor 9 (β = 0.09, *p* = 0.008; *r partial* after controlling for sex and politics = 0.11) were the components of attitude that best predicted this variable: F(3, 626) = 76.61, *p* < 0.001; *R*^2^ *adjusted* = 0.27.

Economic liberalism. Attitude was associated in a significant and inverse way with economic liberalism, whereas political ideology was associated in a significant but positive way with this variable. The size of the effect in this sample was, however, low: (*F*(2, 627) = 22.54, *p* < 0.001; *R*^2^ *adjusted* = 0.06; *r partial* for attitude = −0.17). Factor 9 (β = −0.58, *p* < 0.001; *r partial* after additional control for sex = −0.58), Factor 5 (β = −0.17, *p* = 0.001; *r partial* after controlling for age = −0.22), and Factor 8 (β = 0.16, *p* = 0.001; *r partial* after controlling for age = 0.19) were the components of attitude with a significant relationship with economic liberalism: *F*(4, 625) = 113.04, *p* < 0.001; *R*^2^ *adjusted* = 0.42. 

Limits to economic growth. The global score in attitude and political ideology were also significantly associated with limits to economic growth (*F*(2, 627) = 76.08, *p* < 0.001; *R*^2^ *adjusted* = 0.19; *r partial* for attitude = 0.43). Factor 12 was the best predictor (β = 0.28, *p* < 0.001; *r partial* after controlling for sex and age = 0.31), followed by Factor 4 (β = 0.26, *p* < 0.001) and Factor 3 (β = 0.15, *p* < 0.001): *F*(4, 625) = 55.98, *p* = 0.001; *R*^2^ *adjusted* = 0.26. 

Sustainability. The global score in attitude predicted opinion in sustainability positively and significantly (*F*(1, 628) = 39.24, *p* < 0.001; *R*^2^ *adjusted* = 0.06; β = 0.24), although with a low effect size. According to the partial measures of attitude, Factor 12 (β = 0.19, *p* < 0.001; *r partial* after controlling for age, sex, and politics = 0.19) and Factor 4 (β = 0.19, *p* < 0.001), followed by Factor 8 (β = 0.16, *p* = 0.001; *r partial* after controlling for age = 0.14) and Factor 5 (β = −0.11, *p* = 0.003; *r partial* after controlling for sex and politics = −0.11), constituted the model with the best predictors: *F*(4, 625) = 26.35, *p* = 0.001; *R*^2^ *adjusted* = 0.14.

Social desirability. Although with little predictive capacity, in this sample the relationship between the global value of attitude and desirability reached statistical significance: *F*(1, 628) = 23.01, *p* = 0.001; *R*^2^ *adjusted* = 0.03. The age variable contributed significantly to this model: *F*(2, 627) = 48.92, *p* = 0.001; *R*^2^ *adjusted* = 0.13; *r partial* for attitude = 0.18; *r partial* for age = 0.32. To detail this, Factor 8 (β = 0.22, *p* < 0.001) and Factor 9 (β = 0.19, *p* < 0.001; *r partial* after controlling for sex and politics = 0.19) predicted that score in social desirability: *F*(3, 626) = 51.93, *p* = 0.001; *R*^2^ *adjusted* = 0.20.

It should be noted that social desirability showed a significant correlation with ecological behaviour (*r*^2^ = 0.03, *p* < 0.001) and economic liberalism (*r*^2^ = 0.01, *p* = 0.01). However, this relationship was weak, and the results shown are maintained regardless of the scores in social desirability. 

Altruism. As predictor of altruism we found global attitude (*F*(1, 628) = 79.48, *p* = 0.001; *R*^2^ *adjusted* = 0.11, β = 0.34); but we must highlight the significant effect of social desirability and, to a lesser degree, of the variables age, political tendency, and sex: *F*(5, 624) = 44.24, *p* = 0.001; *R*^2^ *adjusted* = 0.26; *r partial* for attitude = 0.27; *r partial* for social desirability = 0.28). According to the partial measures of attitude, the significant predictors were Factor 4 (β = 0.20, *p* < 0.001), Factor 8 (β = 0.17, *p* < 0.001), Factor 9 (β = 0.10, *p* = 0.006), and Factor 12 (β = −0.09, *p* = 0.02): *F*(7,622) = 41.47, *p* = 0.001; *R*^2^ *adjusted* = 0.31.

## 9. Discussion

This study was designed with the aim of obtaining a multidimensional measure of attitudes applicable in the Spanish context. The starting instrument was the EAI [10] in its abbreviated form with 37 items [22]. This form was created to record environment perception in members of the university community. 

Using this instrument, three consecutive phases were planned, for adaptation to another language. The first one included the translation and a pilot test with university students from the same year. The second consisted of the application of the EAI applied to a wider sample, composed of university students from several years. Although improvable, the findings of the two tests were symmetrical, in terms of reliability and internal structure. The third and last phase perfected the instrument and applied it to a representative sample of the Spanish general population.

The results of the psychometric analysis led us to propose a structure with six first-order factors: environmental movement activism, conservation motivated by anthropocentric concern, confidence in science and technology, personal conservation behaviour, human dominance over nature, and support for population growth policies. Each of the dimensions was represented by three items.

The measurement model was assessed using robust estimation methods. The goodness-of-fit statistics and the individual values of the parameters were acceptable. In line with studies by Milfont and Duckitt [10], the correlations between Factors 3 (environmental movement activism) and 8 (personal conservation behaviour), representative of preservation, were systematically placed among the highest. Although the correlation profile revealed an important degree of independence among the factors underlying the EAI. The internal consistency value for all 18 retained items was nonetheless satisfactory.

As external evidence of validity for the global score and for the different factors, we estimated its convergence with several variables linked to the definition of attitude towards the environment. In Study 1, the overall score in the EAI correlated with the external measurement of neuroticism. This relationship seemed attributable to Factor 12, support for population growth policies, and to a lesser extent in that sample, Factor 5, confidence in science and technology. Factor 12 would be conceptually linked to the superior dimension of preservation in the theory of ecological attitude. Factor 5, however, would be linked to utilisation. 

Findings by Wiseman and Bogner [11] anticipated that high values in neuroticism tended to favour biocentric orientations towards the environment (preservation) and not the anthropocentric (utilisation). Utilisation is, according to Wiseman and Bogner, a general dimension close to inflexibility (tough-mindedness) and egocentrism. Contrarily, preservation would be associated with anxiety, perhaps accompanied by feelings of guilt towards the environment. Several later European studies corroborated the positive relationship between neuroticism and environmental concern [31,32]. Holmström observed, in fact, that attitudes mediated in the relationship between neuroticism and environmental behaviour.

In Study 2 we identified the predicted relationship between the global score in attitude towards the environment and external psychological variables. This relationship was positive and significant with ecological behaviour and limits to economic growth, and negative with economic liberalism. With a broader sample, in study 3 we also found positive associations of the global attitude with sustainability and altruism; although, the size of the effect was larger for ecological behaviour, limits on economic growth, and altruism.

Compared to global attitude, the effect on these external variables was consistently higher when considering partial measurements. According to their conceptual definition, Factor 8 and Factor 3 were the components of attitude that were intensely related to ecological behaviour. Additionally, as expected, Factor 12 (support for population growth policies) was systematically the best predictor of limits to economic growth, and Factor 9 (human dominance over nature) had the highest relative effect on economic liberalism.

On the other hand, we only found significant association between the global score in the EAI and the score in social desirability in Study 3. With regard to the factors, both with university students and in the general population, Factor 8 (personal conservation behaviour) was the main predictor of social desirability. In Study 3, Factor 9 (human dominance over nature) was also associated with this variable. Wiseman and Bogner [11] as well as Milfont and Duckitt [10] pointed out that the utilisation component of attitude would be essentially free of social desirability, while preservation would be affected to a greater extent. Our study revealed that, in any case, the size of the effect did not reach medium, and the scores on social desirability were best explained by the age of the participants.

Irrespective of the aspect of social desirability, the global attitude also correlated positively with the score in altruism. Regarding partial measures, the relative influence on altruism was higher for Factor 8 and for Factor 4 (conservation motivated by anthropocentric concern).

To summarise the above, we can extract the following conclusions on the composition of the EAI:

By factor analysis, we have been able to identify six first-order factors, but not the postulated structure of higher order [3,11,15].

Through external evidence of validity, we obtained some support for the hypothesised link between the preservation factors and the variables of ecological behaviour, limits towards economic growth, sustainability, altruism, and social desirability [10,15,17,18]. Similarly, we reported on the nexus between Factor 9, utilisation, and economic liberalism [10,15].

From the convergence with the external variables, we also deduce that there is an overlap between factors close to preservation and utilisation. It was seen, for example, in the relationship of Factor 4 with altruism. Doubts about discrimination between these possible two higher-order dimensions continue [10].

Another systematic result in Studies 1 and 2 was that we found non-significant relationships between the scores in attitude towards the environment and the variables perceived level of physical health, time dedicated to sports per week, and tobacco consumption. 

With regard to age, Studies 1 and 2 replicated what was obtained with the French version of the EAI [12]. In Study 3, the trend remained: a non-significant relationship between age and global score in attitude and significant, albeit with little predictive power, in the partial measures.

Sex showed no statistically significant effects on the overall score in attitudes towards the environment, although it does in several factors. Sutton and Gyuris [22] had reported differences as regards women in their study with first and last year students from different undergraduate programs. Domingues and Gonçalves [13] found higher scores for women in preservation and lower in utilisation. After the turn of the millennium, research on gender differences in the school environment identified a tendency for women to express greater concern for the natural environment than men. 

This trend was recorded on the levels of attitudes and environmental behaviours and is seen to be stronger among younger students and in those with a female gender orientation or role. However, in general it can be said that this trend was seen regardless of age, country of residence, and even regardless of whether Spanish or English was used [21]. Outside the educational field, results were diverse. Women showed, in general, a better attitude towards the environment, but this attitude was not always reflected in a greater degree of knowledge or more conservative behaviour [5].

Empirical studies aimed at identifying potential determinants of gender difference in environmentalism showed greater socialisation in the case of women [21] and were placed close to the theoretical models based on values [33,34].

Dietz et al. [34] found no discrepancies in the structure of values between women and men yet did in the importance given to those values. Women regarded altruism as more important. Following a similar line of inquiry, Arnocky and Stroink [33] found that the greatest emotional empathy in the case of women contributed to explaining the differences in concern for the environment, as well as in cooperation and competitiveness in sharing ecological resources. 

According to our data, both from students and from a sample of the general Spanish population, women repeatedly showed higher scores than men in the aspect of human dominance over nature (factor associated with utilisation), but lower in support for population growth policies (factor associated to preservation). These results were obtained regardless of age and political tendency. In addition, findings in Study 3 reflected the scant link between sex and the level of altruism. Neither the differences in altruism nor other demographic and personal variables considered led us to conclude that neither women nor men showed a greater concern for the environment.

On the other hand, regardless of sex and age, we find a discrepancy in the attitudes of participants of different political orientation. The participants who were placed on the political right showed a more utilitarian orientation of nature, reflected by their scores in Factor 9, human dominance over nature. According to Moussaoui et al. [12], the scores in attitudes, particularly those of the biocentric type, were also lower for the university students ideologically labelled “right and liberal”, on a scale of political sensibility (left vs. right) and economic (interventionist vs. liberal).

These findings replicate the results of Milfont and Duckitt [10] with the original version of the EAI. From a theoretical perspective, they agree with the traditional association between conservative ideology and dominance over the environment [35], which seem to condition people’s decisions in essential aspects such as energy consumption [36], as well as for those who have explanatory hypotheses such as those connecting political ideology with the moral framework of environmentalism and the intrinsic motivation towards the environment [37,38].

Finally, we found no differences in attitudes towards the environment based on the year that the students belonged to. We also verified that the variation in the results in attitude could not be attributed to the region or participants’ type of place of residence, in contradiction to findings on pro-environment beliefs and behaviours in other cultural contexts ([39,40], among others). As stated by, for example, Shephard [41] or Nejati and Nejati [42], greater environmental commitment in institutions of higher education is important. However, in our context, there are very few environmental education projects. It is, without a doubt, a pending issue. According to Sutton and Gyuris [22], this version of the EAI could be sensitive to differences in attitude as a result of an environmental education program.

Based on the evidence so far, obtained with the first applications of the EAI in Spanish, it should be noted that they offer empirical support for a multidimensional model of attitudes towards the environment. This reduced version of the EAI seems to discriminate between six theoretical dimensions at least, referring both to attitude towards the environment as well as to the more selfish appreciation of nature [43]. A form with 18 items and six conceptual dimensions may leave some facets of the attitudes towards the environment construct uncovered. However, we understand that it is part of the current search for comprehensive models, aimed at predicting behaviour in this field [1,44]. It has the added virtue that response rates are very high, as can be seen in the analyses of this report.

We must admit, however, methodological limitations that the results also show, and which must be paid greater attention to in the future.

On the one hand, the EAI items derived from the theory on environmental attitudes are complex, which can facilitate acquiescence [45]. To prevent this effect, we kept items formulated in a direct and inverse sense, avoided denials, and took the aspect of social desirability into account. On the other hand, the response format used in this version of the EAI complied with the recommendations in the literature regarding the number and meaning of the categories used for testing measuring instruments with university students [46] and obeyed the character of psychometric adaptation of the studies. However, the results were not as expected for six of the dimensions from the original model.

In terms of reliability, the items in the short version we started with were chosen on the basis of item–total correlations and their factorial influence. Therefore, the alpha coefficient is considered to be appropriate for reliability estimations. The scores in the test, however, are expected to represent multiple dimensions, two of which showed modest alpha values, and therefore, other forms of reliability such as test–retest should be considered [47]. 

On the other hand, to ensure the invariance of the items it would be desirable to have delved into its working through cognitive pre-testing [48].

This instrument of measurement in Spanish, developed from the EAI, will certainly benefit from the work with new samples, which should mainly serve the aforementioned effects of measure stability, and also to continue investigating the hierarchical structure of attitude. In this way, we will be able to progress in knowing how people understand their relationship with the environment in our context.

The ultimate objective of this research is to offer a useful tool to measure and monitor change in attitudes. Such a measure contributes to the development of educational programs, investigating the similarities and cultural differences in attitudes towards the environment, and to carrying out research on the link between attitude and sustainable behaviour.

## 10. Conclusions

Regarding both university students and general population samples, our studies reveal an abbreviated model of attitudes towards the environment. The model is internally consistent and allows for six partial measures: environmental movement activism, conservation motivated by anthropocentric concern, confidence in science and technology, personal conservation behaviour, human dominance over nature, and support for population growth policies. 

From the convergence with external measures, we found support for a link between the preservation factors and the variables of ecological behaviour, limits towards economic growth, sustainability, and altruism. Similarly, we described a link between one utilisation factor and economic liberalism. However, the results also showed some overlap between factors close to preservation and utilisation, so questioning the conceptual distance between these possible two higher-order dimensions.

The estimated relations with external variables stayed away from demographic and personal aspects such as age, sex, and political ideology. The effect of social desirability was low, in the expected direction, and in the population sample was best explained by the age of the participants. Among university students, the differences in attitude were independent from perceived level of physical health, time dedicated to sports per week, and tobacco consumption. Finally, the variation in the results in attitude could not be attributed to the region or participants’ type of place of residence.

## Figures and Tables

**Table 1:** Descriptives, confirmatory factor analysis estimations, and internal consistency coefficients for a six-factor model in university students.

		Sample Study 1					Sample Study 2				
Factor	Item	*M*	*SD*	λ_x_	*R* ^2^	α	*M*	*SD*	λ_x_	*R* ^2^	α
Factor 1	Item 01	5.78	1.51	1.19	0.62	0.84	6.18	1.01	0.68	0.44	0.81
	Item 04	5.29	1.54	1.24	0.65		6.00	1.32	1.05	0.63	
	Item 14	6.14	1.08	0.93	0.75		6.43	0.89	0.80	0.79	
Factor 3	Item 03	5.29	1.42	0.93	0.43	0.81	5.00	1.30	0.95	0.53	0.78
	Item 08	4.97	1.34	1.03	0.59		4.63	1.32	0.96	0.53	
	Item 17	5.18	1.23	1.10	0.81		5.04	1.25	0.95	0.57	
Factor 5	Item 06	4.54	1.36	1.19	0.77	0.75	4.34	1.43	1.20	0.72	0.87
	Item 20	3.30	1.49	0.89	0.36		3.57	1.45	1.18	0.67	
	Item 23	4.22	1.17	0.81	0.48		4.33	1.38	1.13	0.67	
Factor 8	Item 26	5.22	1.16	0.70	0.36	0.63	5.12	1.18	0.91	0.59	0.69
	Item 27	6.22	0.79	0.43	0.20		6.16	1.14	0.60	0.27	
	Item 31	6.02	0.83	0.73	0.78		5.88	0.98	0.65	0.44	
Factor 9	Item 24	5.50	1.46	0.99	0.46	0.68	5.95	1.16	0.64	0.30	0.65
	Item 30	6.41	0.98	0.60	0.38		6.32	1.10	0.78	0.50	
	Item 33	5.68	1.69	1.16	0.47		5.88	1.37	0.86	0.39	
Factor 12	Item 11	2.57	1.60	1.09	0.46	0.76	2.44	1.44	1.31	0.82	0.76
	Item 29	2.49	1.50	1.60	1.14		2.43	1.43	1.12	0.61	
	Item 37	3.47	1.58	0.72	0.21		3.36	1.67	0.87	0.27	

*Note.* Sample Study 1 = pre-test (*n* = 125); Sample Study 2 = test (*n* = 206). Factor 1 = enjoyment of nature; Factor 3 = environmental movement activism; Factor 5 = confidence in science and technology; Factor 8 = personal conservation behaviour; Factor 9 = human dominance over nature; Factor 12 = support for population growth policies.

**Table 2:** Significant regression coefficients of external variables on attitudes in university students (*n* = 206).

External Variable	Significant Predictors	*F*	*Adjusted R* ^2^	*Beta*
Ecological behaviour	Attitude overall, age	26.32	0.20	0.40, −0.18
	Factor 8, Factor 3, Factor 5, Age	42.77	0.45	0.50, 0.22, −0.12, −0.16
Economic liberalism	Attitude overall, politics	20.62	0.16	−0.35, 0.18
	Factor 9, Factor 3, politics	16.37	0.18	−0.31, −0.19, 0.15
Limits economic growth	Attitude overall, politics	15.11	0.12	0.32, −0.13
	Factor 12, politics	9.68	0.08	0.24, −0.17
Social desirability	Factor 8	6.79	0.01	0.18

*Note.* Factor 3 = environmental movement activism; Factor 5 = confidence in science and technology; Factor 8 = personal conservation behaviour; Factor 9 = human dominance over nature; Factor 12 = support for population growth policies. All regressions were checked for multicollinearity. Tolerance values fell between 0.74 and 1.

**Table 3:** Descriptives, confirmatory factor analysis estimations, and internal consistency coefficients for a six-factor model in general population (*n* = 630).

Factor	Item	*M*	*SD*	λ_x_	*R^2^*	α
Factor 3	Item 03	4.89	1.46	1.06	0.53	0.80
	Item 08	4.80	1.46	1.08	0.55	
	Item 17	4.74	1.48	1.21	0.67	
Factor 4	Item 02	5.67	1.40	0.84	0.36	0.64
	Item 07	6.08	1.34	0.74	0.31	
	Item 18	5.07	1.45	0.97	0.45	
Factor 5	Item 06	3.94	1.68	1.05	0.39	0.64
	Item 20	4.13	1.73	0.41	0.06	
	Item 23	4.13	1.73	1.80	1.08	
Factor 8	Item 26	5.34	1.27	1.00	0.62	0.81
	Item 27	6.05	1.25	0.93	0.55	
	Item 31	5.80	1.19	0.92	0.60	
Factor 9	Item 24	4.72	1.83	1.44	0.61	0.80
	Item 30	5.19	1.83	1.43	0.61	
	Item 33	4.43	1.89	1.34	0.50	
Factor 12	Item 11	3.51	1.90	1.09	0.71	0.85
	Item 29	3.35	1.94	1.60	0.84	
	Item 37	3.80	1.73	0.72	0.45	

*Note.* Factor 3 = environmental movement activism; Factor 4 = conservation motivated by anthropocentric concern; Factor 5 = confidence in science and technology; Factor 8 = personal conservation behaviour; Factor 9 = human dominance over nature; Factor 12 = support for population growth policies.

**Table 4:** Significant regression coefficients of external variables on attitudes in general population (*n* = 630).

External Variable	Significant Predictors	*F*	*Adjusted R* ^2^	*Beta*
Ecological behaviour	Attitude overall, age	65.53	0.17	0.41, 0.08
	Factor 8, Factor 3, Factor 9	76.61	0.27	0.33, 0.25, 0.09
Economic liberalism	Attitude overall, politics	22.54	0.06	−0.17, 0.16
	Factor 9, Factor 5, Factor 8, politics	113.04	0.42	−0.58, −0.17, 0.16
Limits economic growth	Attitude overall, politics	76.08	0.19	0.43, −0.04
	Factor 12, Factor 4, Factor 3, politics	55.98	0.26	0.28, 0.26, 0.15, −0.07
Sustainability	Attitude overall	39.24	0.06	0.24
	Factor 12, Factor 4, Factor 8, Factor 5	26.35	0.14	0.19, 0.19, 0.16, −0.11
Social desirability	Attitude overall, age	48.92	0.13	0.18, 0.32
	Factor 8, Factor 9, age	51.93	0.20	0.22, 0.19, 0.28
Altruism	Attitude, social d., age, politics, sex	44.24	0.26	0.25, 0.27, 0.16, −0.12, 0.07
	Factor 4, Factor 8, Factor 9, Factor 12,	47.76	0.23	0.20, 0.17, 0.10, −0.09
	social d., age, politics			0.22, 0.14, −0.11

*Note.* Social d. = social desirability. Factor 3 = environmental movement activism; Factor 4 = conservation motivated by anthropocentric concern; Factor 5 = confidence in science and technology; Factor 8 = personal conservation behaviour; Factor 9 = human dominance over nature; Factor 12 = support for population growth policies. All regressions were checked for multicollinearity. Tolerance values fell between 0.66 and 0.998.

## Data Availability

Data files will be available from the MINERVA Institutional Repository (accession number(s) to be assigned).

## References

[1] Gifford R. (2014). Environmental psychology matters. Annu. Rev. Psychol..

[2] Kaiser F.G., Schultz P.W. (2009). The attitude-behavior relationship: A test of three models of the moderating role of behavioral difficulty. J. Appl. Soc. Psychol..

[3] Johnson B., Manoli C.C. (2010). The 2-MEV scale in the United States: A measure of children’s environmental attitudes based on the theory of ecological attitude. J. Environ. Educ..

[4] Milfont T.L. (2009). A functional approach to the study of environmental attitudes. Psyecology.

[5] Gifford R., Sussman R., Clayton S.D. (2012). Environmental attitudes. The Oxford Handbook of Environmental and Conservation Psychology.

[6] Hawcroft L.J., Milfont T.L. (2010). The use (and abuse) of the new environmental paradigm scale over the last 30 years: A meta-analysis. J. Environ. Psychol..

[7] McIntyre A., Milfont T.L. (2016). Who cares? Measuring environmental attitudes. Research Methods for Environmental Psychology.

[8] Bogner F.X., Wilhelm M.G. (1996). Environmental perception of pupils: Development of an attitude and behaviour scale. Environmentalist.

[9] Dunlap R.E., Van Liere K.D., Mertig A.G., Jones R.E. (2000). Measuring endorsement of the new ecological paradigm: A revised NEP scale. J. Soc. Issues.

[10] Milfont T.L., Duckitt J. (2010). The environmental attitudes inventory: A valid and reliable measure to assess the structure of environmental attitudes. J. Environ. Psychol..

[11] Wiseman M., Bogner F.X. (2003). A higher-order model of ecological values and its relationship to personality. Pers. Individ. Differ..

[12] Moussaoui L.S., Desrichard O., Mella N., Blum A., Cantarella M., Clémence A. (2016). French validation of the Environmental Attitudes Inventory. Eur. Rev. Appl. Psychol..

[13] Domingues R.B., Gonçalves G. (2018). Assessing environmental attitudes in Portugal using a new short version of the Environmental Attitudes Inventory. Curr. Psychol..

[14] Rahim N., Apendi S.R.M., Farook F., Ismail A., Radzi S.M., Hanafiah M.H.M., Sumarjan N., Mohi Z., Sukyadi D., Suryadi K., Purnawarman P. (2016). Environmental Attitudes Inventory (EAI) of UiTM Penang hospitality stu-dents. Heritage, Culture and Society.

[15] Milfont T.L., Duckitt J. (2004). The structure of environmental attitudes: A first- and second-order confirmatory factor analysis. J. Environ. Psychol..

[16] De Groot J.I.M., Steg L. (2007). Value orientations to explain beliefs related to environmental significant behavior: How to measure egoistic, altruistic, and biospheric value orientations. Environ. Behav..

[17] Milfont T.L., Sibley C.G., Duckitt J. (2009). Testing the moderating role of the components of norm activation on the relationship between values and environmental behavior. J. Cross-Cult. Psychol..

[18] Schultz W.P., Gouveia V.V., Cameron L.D., Tankha G., Schmuck P., Franek M. (2005). Values and their relationship to environ-mental concern and conservation behavior. J. Cross Cult. Psychol..

[19] Cotton D.R.E., Alcock I. (2013). Commitment to environmental sustainability in the UK student population. Stud. High Educ..

[20] Liefländer A.K., Bogner F.X. (2014). The effects of children’s age and sex on acquiring pro-environmental attitudes through environmental education. J. Environ. Educ..

[21] Zelezny L.C., Chua P.-P., Aldrich C. (2000). Elaborating on gender differences in environmentalism. J. Soc. Issues.

[22] Sutton S.G., Gyuris E. (2015). Optimizing the environmental attitudes inventory. Int. J. Sustain. High. Educ..

[23] Cordero A., Pamos A., Seisdedos N. (2008). Inventario de personalidad NEO-PI Revisado.

[24] Satorra A., Bentler P.M., von Eye A., Clogg C.C. (1994). Corrections to test statistics and standard errors in covariance structure analysis. Latent Variables Analysis: Applications for Developmental Research.

[25] Schultz P.W., Zelezny L., Dalrymple N.J. (2000). A multinational perspective on the relation between judeo-christian religious beliefs and attitudes of environmental concern. Environ. Behav..

[26] Kilbourne W.E., Beckmann S.C., Thelen E. (2002). The role of the dominant social paradigm in environmental attitudes: A mul-ti-national examination. J. Bus Res..

[27] World Commission on Environment and Development (1987). Our Common Future.

[28] Moral de la Rubia J., García-Cadena C.H., Antona-Casas C.J. (2012). Translation and validation of the Balanced Inventory of desirable responding in a probability sample of Mexican university students. Rev. Psicol. GEPU.

[29] Mikulic I.M., Crespi M., Caballero R. (2016). Psychometric study of Balanced Inventory of Desirable Responding. Anu. Psicol..

[30] Rey P. (2004). Methodological note on the CIS’ barometer indicators. REIS Rev. Invest. Sociol..

[31] Hirsh J.B. (2010). Personality and environmental concern. J. Environ. Psychol..

[32] Holmström S.J.N. (2015). The Influence of Neuroticism on Proenvironmental Behavior. Bachelor’s Thesis.

[33] Arnocky S., Stroink M. (2011). Gender differences in environmentalism: The mediating role of emotional empathy. CRISP.

[34] Dietz T., Kalof L., Stern P.C. (2002). Gender, values, and environmentalism. Soc. Sci. Q..

[35] Feygina I. (2013). Social justice and the human–environment relationship: Common systemic, ideological, and psychological roots and processes. Soc. Justice Res..

[36] Gromet D.M., Kunreuther H., Larrick R.P. (2013). Political ideology affects energy-efficiency attitudes and choices. Proc. Natl. Acad. Sci. USA.

[37] Feinberg M., Willer R. (2012). The moral roots of environmental attitudes. Psychol. Sci..

[38] Sherman A.K., Rowe A.J., Bird S., Powers S., Leagult L. (2016). Motivational orientation explains the link between political ideology and conservation efforts. Ecopsychology.

[39] Dean A.J., Fielding K.S., Newton F.J. (2016). Community knowledge about water: Who has better knowledge and is this associated with water-related behaviors and support for water-related policies?. PLoS ONE.

[40] Milfont T.L., Evans L., Sibley C.G., Ries J., Cunningham A. (2014). Proximity to coast is linked to climate change belief. PLoS ONE.

[41] Shephard K. (2010). Higher education’s role in ‘education for sustainability’. AUR.

[42] Nejati M., Nejati M. (2013). Assessment of sustainable university factors from the perspective of university students. J. Clean. Prod..

[43] Kaiser F.G., Hartig T., Brügger A., Duvier C. (2011). Environmental protection and nature as distinct attitudinal objects: An ap-plication of the Campbell Paradigm. Environ. Behav..

[44] Kormos C., Gifford R. (2014). The validity of self-report measures of proenvironmental behavior: A meta-analytic review. J. Environ. Psychol..

[45] Krosnick J.A., Presser S., Marsden P.V., Wright J.D. (2010). Question and questionnaire design. Handbook of Survey Research.

[46] Weijters B., Cabooter E., Schillewaert N. (2010). The effect of rating scale format on response styles: The number of response cate-gories and response category labels. Int. J. Res. Mark..

[47] Danner D., Blasius J., Breyer B., Eifler S., Menold N., Paulhus D.L., Rammstedt B., Roberts R.D., Schmitt M., Ziegler M. (2016). Current challenges, new developments, and future directions in scale construction. Eur. J. Psychol. Assess..

[48] Lenzner T., Neuert C., Otto W. (2016). Cognitive Pretesting. GESIS Survey Guidelines.

